# Geminated Maxillary Incisors With Talon Cusp: A Case Report With Management and Narrative Review

**DOI:** 10.1155/crid/5856963

**Published:** 2026-11-15

**Authors:** Mohammed Sultan Al-Ak′hali, Reem Hassan Kelani, Rahaf Ahmed Halawi, Hani Saeed Alakhali, Atyaf Fassal Abueishah, Syed Wali Peeran

**Affiliations:** ^1^ Department of Preventive Dental Sciences College of Dentistry Jazan University Jazan Saudi Arabia jazanu.edu.sa; ^2^ Dental Department Saudi German Hospital SGH Khamis Mushait Saudi Arabia

**Keywords:** case report, geminated maxillary incisors, talon cusp

## Abstract

**Background and Objectives:**

Recognition of teeth with gemination and/or talon cusp, along with early treatment, is important to prevent complications such as occlusal interference, malocclusion, the formation of caries and periodontal pockets, and esthetic issues. This case report is aimed at describing the multidisciplinary management of a rare case of geminated permanent maxillary central incisors associated with a talon cusp and to review similar cases previously reported in the literature.

**Materials and Methods:**

The diagnoses were determined through a combination of intraoral photographs, dental casts, periapical x‐rays, and cone‐beam computed tomography. A long‐term, multidisciplinary treatment plan was developed for managing this geminated tooth with a talon cusp.

**Results:**

This case report describes the successful diagnosis and management of an 11‐year‐old patient with Class II malocclusion and bilateral gemination of the two maxillary incisors. A unilateral talon cusp was erupting on the palatal surface of the malpositioned maxillary left central incisor, extending from the cervical area toward the incisal edge and reaching approximately two‐thirds of the crown length. The talon cusp caused occlusal interference with the mandibular left lateral incisor, and a 5‐mm periodontal pocket had formed at the distopalatal site of the maxillary left central incisor. The treatment focused on managing tooth gemination through a combination of occlusal adjustment, restorative procedures, and orthodontic therapy. The talon cusp was gradually reduced in size through selective enamel grinding to eliminate occlusal interference, followed by the application of topical fluoride. Teeth affected by caries were restored with appropriate fillings, and orthodontic treatment was provided as necessary.

**Conclusion:**

The rehabilitation of geminated maxillary anterior incisors with a talon cusp requires a multidisciplinary approach for optimal management and outcomes.

## 1. Introduction

Gemination is a rare dental anomaly. The etiology is unknown; it appears that gemination is caused by composite interactions among a variation of environmental and genetic factors. Some reports mention that it may be caused by the unsuccessful attempt of a single tooth bud to form two teeth. It developed from differentiation stage of teeth development [1]. Gemination causes esthetic complications, malposition, and impaction of adjacent teeth because of the greater size of the geminated teeth crowns [2]. The prevalence of geminated teeth ranges from 0.07% to 2.1% [2–4]. They are more common in deciduous teeth (about 1%) than in permanent teeth (about 0.1%), especially in maxillary deciduous canines and incisors [2]. Upper central incisors are most frequently affected by gemination [3, 4]. Talon cusp has been named with various terms such as cusped cingulum, dens evaginatus, supernumerary cusp, supernumerary lingual tubercle, and accessory cusp and hyperplastic cingulum [5]. The conventional definition of talon cusp considers them as an accessory cusp projecting palatally/lingually and facial/labial from the cingulum to the incisal edge [6]. The causes seem to be multifactorial with a possible chance of genetic participation routing to disorders in teeth development at the morphodifferentiation stage. A few number of talon cusp cases among the family members have been published, which fully supports the genetic influence [6, 7]. Talon cusp consists of enamel, dentin, and a varying amount of pulp tissue [8]. The precise etiology of talon cusp is controversial. It may be caused by environmental and genetic factors, altered endocrine functions, a transient focal hyperplasia of the mesenchymal dental papilla, outward folding of the inner enamel epithelium, and an aberrant hyperactivity of dental lamina [8, 9]. There is a suggestion of a strong genetic impact in its formation as evidenced by its occurrence in close family partners [10]. Typically, a single tooth is affected by talon cusp; however, instances of multiple or bilateral involvement are not uncommon. It has been reported to occur in males more than females [8, 11]. It is more common in the permanent teeth (75%) than in the deciduous teeth [12, 13]; it has been reported to occur more often in permanent anterior teeth of the upper jaw (92%) than in those of the lower jaw [12–14]. The maxillary central incisor is the most affected in the deciduous teeth, whereas the maxillary lateral incisor is the most frequently affected in the permanent teeth [12]. Talon cusps vary widely in shape, size, structure, location and origin but often found on the palatal surfaces of the dentation; a few reports found on the facial surfaces of the dentation [15–20] and on supernumerary teeth [21]. This anomaly may happen as solitary anomaly or with other dental anomalies such as supernumeraries, complex odontomas, shovel‐shaped incisors, bifid cingulum, unerupted canines, the large cusp of Carabelli, dens invaginatus, and peg‐shaped lateral incisors [13, 22, 23]. Clinical complications that may result from presence of big prominent cusps may cause clinical problems, including occlusal interference and possibility of temporomandibular joint pain, displacement of the affected tooth, periodontal pockets, carious lesions in the developmental grooves and pulpal exposure due to cuspal attrition, cuspal fracture, pulpal necrosis, periapical pathoses, and tongue irritation during mastication or speech [22, 24, 25]. Some cases may have esthetic complaints because of the inclination or rotation of the tooth associated with the talon cusp. This is also true in patients with labial talon′s cusp [22]. A talon cusp can cause occlusal interference, leading to periodontal issues [2]. Sex and talon cusp were not statistically significantly associated [26]. Talon cusps were detected in 44 teeth out of a total of 35,424 teeth to give a tooth prevalence of 0.12% [27].

The prevalence of talon cusp varies considerably among different ethnic populations, with reported rates ranging from 0.06% to 7.7%. Studies have documented prevalence rates of 0.06% in Mexican populations, 0.17% in American populations, 0.19% to 0.58% in Indian populations, 1.2% in Turkish populations, 2.4% in Jordanian populations, 2.5% in Hungarian populations, 5.2% in Malaysian populations, and up to 7.7% in North Indian populations [27–34].

A few studies investigate the talon cusp with various discrepancies in clinical presentations. The use of precise diagnostic criteria in the classification of talon cusps and consistent reporting will assist in determining the existing prevalence rate. This approach will help mitigate issues such as occlusal disharmony, periodontal diseases, caries, periapical pathology, and esthetic concerns. Classification of talon cusps used in this study was based on the degree of extension and formation according to Hattab et al. [13] who diagnosed three types of talon cusps: (i) true talon (an accessory cusp that obviously projects from the lingual or palatal surface of a permanent or primary anterior teeth and extends at least half the distance from the cementoenamel junction (CEJ) to the incisal edge), (ii) semitalon (an accessory cusp of 1 mm or more but extending less than the distance from the CEJ to the incisal edge), and (iii) trace talon (enlarged or prominent cingulum and variations, i.e., conical, bifid, or tubercle‐like). Although a talon cusp may occur as an isolated dental anomaly, it can also be associated with other crown abnormalities, including supernumerary teeth, dens invaginatus, and peg‐shaped crowns. In very rare cases, it has also been reported in association with geminated teeth. [13, 15, 23, 27–29]. Multidisciplinary approach should apply for the management of geminated teeth with talon cusp using restorative, orthodontic and sometimes may require extraction of geminated lateral incisors and reshape the canine after complete eruption to the shape of lateral incisor followed by the alignment of anterior teeth [30]. Talon cusps can be managed by selectively grinding the enamel surface until the interference is eliminated. If this reduction causes pulpal exposure, appropriate endodontic treatment should be completed. This study is aimed at introducing a multidisciplinary management of a rare case of geminated permanent maxillary central incisors associated with talon cusp and review on previous reported cases.

## 2. Case Presentation

The study was approved by the Scientific Research Ethics Committee of Jazan University (Approval Number REC 45/10/1065) and was carried out in compliance with the Helsinki Declaration on medical research involving human subjects.

An 11‐year‐old male patient, accompanied by his father, presented to the dental clinic at the College of Dentistry, Jazan University, seeking orthodontic treatment.

The chief complaint was a large upper anterior tooth with an additional smaller tooth‐like structure located behind the upper left central incisor. This anomaly was causing tongue irritation and discomfort due to contact with a lower tooth (Figure 1a,c). A comprehensive general examination was conducted to rule out any associated systemic diseases or syndromes. The patient appeared healthy and exhibited normal physical development for his age. There was no history of orofacial trauma, no evidence of any syndromic conditions, and no similar cases reported within the family, although the parents are first cousins, indicating consanguinity.

**Figure 1:**
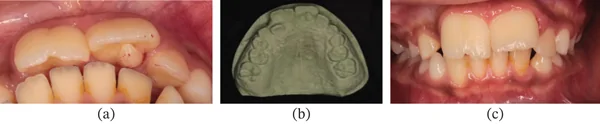
(a) Clinical photograph and occlusal view of maxillary permanent central incisors with gemination anomaly. Talon cusps on the palatal surface of the left maxillary central incisor. (b) Dental cast showing the maxillary arch with geminated maxillary central incisors and associated palatal talon cusp. (c) Clinical photograph of the teeth from the facial aspect.

Intraoral examination revealed a Class II Division 1 incisor relationship on a Class I skeletal base with average vertical proportions. The overjet measured 5.5 mm to Tooth 21, with crowding noted in the upper anterior region. The maxillary central incisors presented with enlarged, bifid crowns, with a prominent talon cusp located on the palatal aspect of the left central incisor. This anomaly caused palatal displacement of the right maxillary lateral incisor. Clinically, the talon cusp was continuous with the crown structure and was separated from the palatal surface of the crown by distinct developmental grooves on either side. Careful clinical examination showed that these developmental grooves were noncarious and free of discoloration or signs of decay. The talon cusp was composed of normal enamel and dentin and demonstrated a clear continuity with the crown morphology.

The enlarged crowns, resulting from gemination, contributed to anterior crowding and reduced the available space for proper alignment of the right lateral incisor (Figure 1b). The diagnosis of gemination was established based on both the clinical and cone‐beam computed tomography (CBCT) findings. Clinically, the maxillary central incisors exhibited enlarged bifid crowns with a developmental groove extending incisally, a characteristic feature of an attempted division of a single tooth germ. CBCT examination demonstrated that the affected left maxillary central incisor possessed a single root with two root canals that converged in the middle third of the root and terminated through a common apical foramen. In addition, the crown exhibited three pulpal horns corresponding to the bifid crown morphology. These findings support the interpretation of partial cleavage of a single tooth germ rather than the union of two independent tooth germs. Fusion was considered in the differential diagnosis; however, fusion typically results in the union of two separate developing teeth and is often associated with either two distinct roots, two separate pulp chambers, or a more complete separation of the root canal systems. Furthermore, no radiographic evidence of an additional tooth structure or supernumerary component was identified on CBCT, making fusion with a supernumerary tooth unlikely. Twinning was also excluded because complete division of the tooth germ, resulting in the formation of two identical teeth, was not present. Therefore, the combination of a bifid enlarged crown, a predominantly single‐rooted morphology, and a connected canal system was considered most consistent with incomplete gemination. The enlarged crown dimensions contributed to a reduction in arch space, resulting in anterior crowding and palatal displacement of the adjacent right maxillary lateral incisor.

The mesiodistal widths of the left and right maxillary central incisors measured 15 and 12 mm, respectively, whereas their cervico‐incisal dimensions measured 14 and 11 mm. A well‐defined, pyramidal‐shaped talon cusp was present on the maxillary left central incisor, arising from the distopalatal and midpalatal aspects of the crown. The cusp measured 6 mm in length, extending more than halfway from the CEJ to the incisal edge. Occlusal interference was observed between the talon cusp and the mandibular left lateral incisor, confirmed using thin articulating paper (12 or 20 microns), which showed colored markings indicating points of contact (Figure 1a). Due to the interference during mastication, the maxillary left central incisor exhibited labial tipping. The width of the talon cusp tapered from 5 mm at the cervical area to 2 mm at its occlusal end (Figure 2). Intraoral periapical radiography and CBCT were performed to confirm the clinical findings. Both maxillary left central incisors responded positively to electric pulp testing, indicating vitality. The periapical radiograph revealed a V‐shaped radiopaque structure over the crown of the maxillary left central incisor, consistent with a talon cusp (Figure 3a). CBCT imaging showed a cusp arising from the palatal surface of the left maxillary permanent central incisor, containing three pulpal horns (Figure 3b). Furthermore, the CBCT revealed bifurcation of the pulp chamber, with distinct buccal and palatal pulp horns, the latter extending into the talon cusp (Figure 4). Based on the clinical and radiographic features, a definitive diagnosis of nonsyndromic talon cusp Type I was made, according to the classification by Hattab et al. [13]. The maxillary central incisors were fully developed, with closed apices and no signs of caries or periapical pathology. Both teeth responded positively to thermal and electric pulp sensitivity tests, indicating vitality. Periodontal probing of the maxillary left central incisor with the talon cusp was performed using a Williams probe. A probing depth of 5 mm was recorded at the distopalatal site (Figure 2a), whereas all other sites showed normal sulcus depths—2 mm at the midbuccal and midpalatal sites (Figure 2b), and 2 mm at the mesiobuccal and mesiopalatal sites (Figure 2c). Following a comprehensive clinical and radiographic assessment, the most appropriate treatment plan for managing the geminated permanent maxillary central incisors with an associated talon cusp was established. Various treatment options were presented to the patient′s parent and discussed in detail. These included (1) no immediate treatment with periodic clinical and radiographic monitoring, which would avoid unnecessary intervention but may allow the development of esthetic, functional, or occlusal complications; (2) selective reduction of the talon cusp with topical fluoride application, which could improve occlusion and esthetics while carrying a risk of dentin exposure, tooth sensitivity, or pulpal irritation; (3) endodontic treatment if pulp involvement occurred during cusp reduction or if pulpal pathology developed, providing symptom relief and preservation of the tooth but involving a more invasive procedure; and (4) extraction of the affected tooth followed by appropriate prosthetic or orthodontic management, which would eliminate the anomaly but result in tooth loss and the need for additional treatment. The potential risks, benefits, prognosis, and expected outcomes of each option were thoroughly explained, and informed consent for the selected treatment was obtained from the patient′s parent. Informed consent was obtained, including consent for the use of clinical images. The consent form for the minor was signed by his father. Talon cusp reduction was carried out gradually using a water‐cooled diamond bur mounted on a high‐speed handpiece. The reduction was performed at 8‐week intervals over the course of five consecutive appointments [31] and fluoride application (Fluocal Soluté) was performed for 5 minutes to reduce teeth hypersensitivity after treatment. The talon cusp was gradually ground down to encourage more reparative dentin formation, rather than just reducing the incisal tip. This approach was chosen because odontoblasts are primarily distributed along the full length of the talon cusp [25]. The shape and size of the geminated central incisors were refined using conservative direct composite resin restorations to improve crown morphology, esthetics, and symmetry while preserving maximum tooth structure (Figure 5b). At the second treatment visit, conducted 4 weeks after the initial appointment, a reduction in the periodontal pocket depth at the distopalatal site of the maxillary left central incisor—adjacent to the talon cusp—was observed, decreasing from 5 to 4 mm (Figure 5b) [32]. At the third treatment visit, 3 months after the initial appointment, the periodontal pocket depth had further reduced to 2 mm (Figure 5c). Removable orthodontic treatment was initiated to reposition the left maxillary lateral incisor. The patient was placed on a follow‐up schedule and recalled for maintenance visits at 1, 3, 6, and 12 months. During these visits, the teeth remained asymptomatic, showed no occlusal interference with the lower teeth, and exhibited no signs of periapical pathology. They continued to respond positively to pulp sensitivity tests. The odontoplasty protocol was performed independently of the periodontal follow‐up visits. The gradual reduction and reshaping of the geminated maxillary central incisors and associated talon cusp were carried out over five consecutive appointments at 8‐week intervals.

**Figure 2:**
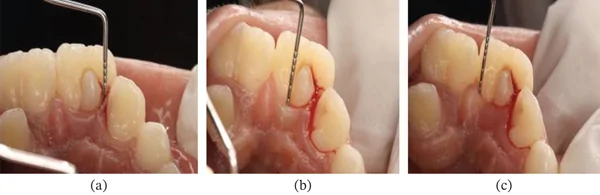
(a) Preoperative palatal view shows 5‐mm pocket depth in the distopalatal site of the maxillary left central incisor. (b) Preoperative 2‐mm gingival sulcus in the midpalatal site. (c) Preoperative 2‐mm gingival sulcus in the mesiopalatal site.

**Figure 3:**
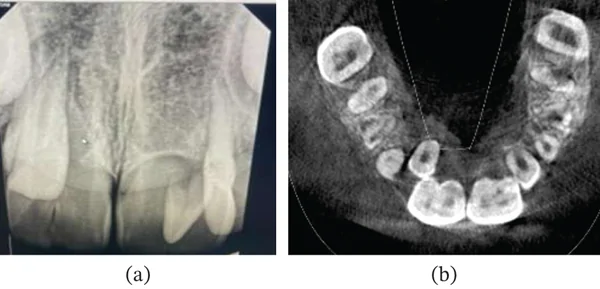
(a) Periapical radiograph of the upper anterior region revealed the presence of a V‐shaped radiopaque structure superimposed on the left maxillary permanent central incisor. (b) CBCT shows a talon cusp projecting from the palatal surface of the left maxillary permanent central incisor with three pulpal horns.

**Figure 4:**
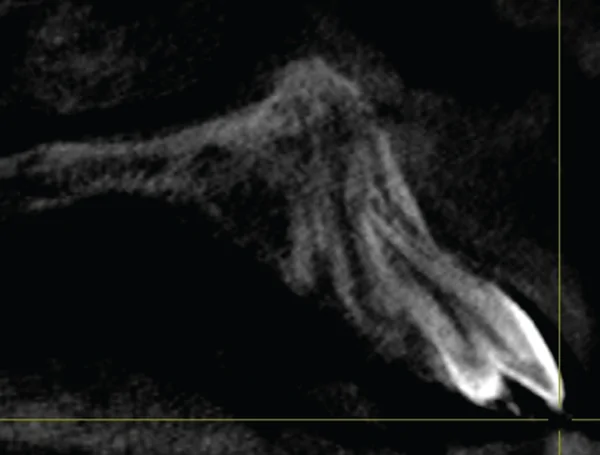
Cone‐beam computed tomography (CBCT) clearly demonstrated the bifurcation of the pulp chamber of maxillary left central incisor, with a buccal and palatal pulp horn, which extended into the talon cusp.

**Figure 5:**
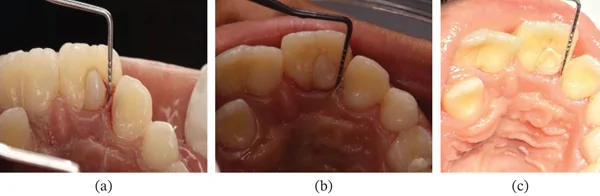
(a) Preoperative 1st‐visit palatal view shows bleeding on probing and 5‐mm pocket depth in the distopalatal site of the maxillary left central incisor. (b) Postoperative 2nd‐visit (after 1 month) 4‐mm pocket depth in the distopalatal site of the maxillary left central incisor without bleeding on probing. (c) Postoperative 3rd‐visit (after 3 months) 2‐mm gingival sulcus in the distopalatal site of the maxillary left central incisor without bleeding on probing.

## 3. Discussion

Bilateral gemination of the permanent maxillary central incisors is a rare occurrence, and its association with a talon cusp is even more uncommon. To date, only four cases have been reported in the literature Table 1. Therefore, this case represents the fifth documented instance of a talon cusp associated with bilaterally geminated maxillary central incisors. Encountering rare dental anomalies poses a significant challenge for dentists, as accurate diagnosis and appropriate treatment are often more complex in such cases. Therefore, early identification of uncommon anomalies such as the presence of a talon cusp in either primary or permanent geminated teeth is crucial. Understanding the diagnostic methods and treatment options for gemination and talon cusps can help prevent complications including occlusal disharmony, periodontal disease, dental caries, periapical pathology, and esthetic concerns. Timely intervention can reduce the risk of adverse clinical outcomes and enhance patient well‐being. Most studies on geminated teeth and talon cusps have been presented as case reports, primarily addressing issues such as occlusal interference, malocclusion, tooth misalignment, esthetic concerns, and caries formation [23, 35–37]. Both anomalies can lead to various clinical complications. Gemination may result in malpositioned teeth, impaction of adjacent teeth, and compromised esthetics due to the enlarged crown size [2]. A talon cusp can contribute to occlusal interference, tongue irritation, increased susceptibility to caries, esthetic issues, and the development of periodontal problems [2]. Additionally, the geminated left central incisor in this case has three pulpal horns and two root canals that converge in the middle third of the root, whereas geminated incisors typically have a single large pulp chamber and root canal, as described in the literature [23]. The talon cusp consists of enamel and dentin and may contain an extension of pulp tissue, as noted in most previous case reports (Table 1). In some instances, the talon cusp may separate from the crown, increasing the linguolabial width [33]. This separation creates a groove or fissure between the tooth and the talon cusp, making the area susceptible to caries and potentially leading to pulpal complications [33].

**Table 1:** Clinical details and history of all documented cases of talon cusp on a geminated tooth.

Reported cases author (year)	Age of the patient (year) and gender	Unilateral/bilateral geminated tooth	FDI notation affected tooth nos.	Width of tooth	Width of talon cusp	Length of talon cusp	Pulp extension into talon cusp	Associate with periodontal disease
Cullen and Pangrazio (1985)	8 male	Bilateral	11	12 mm	Not stated	Not stated	Yes	Not stated
Al‐Omari et al. (1999)	8 female	Unilateral	11	10.4 mm	4.1 mm	5.5 mm	Yes	Not stated
Gunduz and Acıkgoz [38]	11 male	Unilateral	11	Not stated	4 mm	3.1 mm	No	Not stated
Tomazino et al. [39]	28 female	Unilateral	11	Not stated	Not stated	Not stated	Not stated	Not stated
Hattab and Hazzaa (2001)	9 male	Unilateral	21	11.1 mm	3.3 mm	4.5 mm	Yes	Yes
Sevgi Sener et al. [40]	17 male	Bilateral	#11–#21	12.1–12.7 mm	4.3–4.7 mm	5.6 mm 5.9 mm	Yes	Not stated
Neeraja and Kayal [41]	10 male	Unilateral	32	Not stated	4 mm	6 mm	Yes	Not stated
Castelino [42]	24 male	Unilateral	31	Not stated	Not stated	Not stated	Not stated	Yes
Cubukcu et al. [43]	13 female	Unilateral	21	15 mm	3 mm	9 mm 8 mm	Yes	Yes
Nandini et al. (2021)	23 female	Bilateral	#13–#23	Not stated	Not stated	Not stated	Not stated	Not stated
Archna Agnihotri et al. [44]	12 male	Unilateral	11	Not stated	Not stated	Not stated	No	Not stated
Tarım Ertaş et al. [45]	17 male	Bilateral	#11–#21	Not state	Not state	Not stated	Yes	Not stated
Present case	11 male	Bilateral	#11–#21	15 mm 12 mm	5 mm	6‐mm	Yes	Yes

Several studies suggest that the anatomical structure of the talon cusp can cause occlusal interference, which may result in periodontal damage due to occlusal trauma [23, 35, 36, 46]. However, the development of periodontal pockets associated with these anomalies is rare and has been poorly documented. Among 12 case reports reviewed (see Table 1), nine did not report measurements of pocket depth related to geminated teeth or talon cusps. Only three reports have indicated a connection between periodontal disease and the presence of a talon cusp on a geminated tooth. Our case represents the fourth documented instance of periodontal pocket formation around a geminated tooth with a talon cusp. A periodontal pocket was observed between the distal aspect of the talon cusp and the interproximal area of the left central and lateral incisors (Figure 5a). The talon cusp obliterated the interproximal space, impeding effective oral hygiene measures and creating a plaque‐retentive niche that contributed to periodontal pocket development (Figure 5).

The localized periodontal pocket was not attributed solely to occlusal interference. In the present case, the prominent talon cusp obliterated the interproximal space between the affected teeth, creating a plaque‐retentive area that hindered effective oral hygiene and promoted localized periodontal inflammation and pocket formation. Gradual reduction of the talon cusp improved access for plaque control, eliminated the plaque‐retentive niche, and facilitated periodontal healing, which was reflected in the reduction of the pocket depth to within the normal range.

This case report highlights two dental anomalies a large geminated tooth with fissures and a prominent Type I talon cusp, both of which may predispose the patient to periodontal disease. The evaluation of periodontal pocket formation in this study indicates that it was associated with occlusal interference. However, only one report Hegde and Ashok Kumar [29] has suggested that periodontal pockets can sometimes develop even in the absence of occlusal interference. These findings indicate that the subgingival extension of a talon cusp itself may act as a local risk factor for periodontal destruction. Managing a geminated tooth with a talon cusp as early as possible is essential to prevent clinical complications and their consequences. No treatment, pit and fissure sealants, pulp therapy, gradual grinding of the enamel surface of the talon cusp followed by a partial pulpotomy [19], restorative therapy [20] are all options for managing the talon cusp; in some advanced cases, complete removal of the cusp is indicated along with pulp extirpation and root canal therapy [25]. Few cases of talon cusp therapy have been documented in the literature using different types of treatments as follows: in a case series including the management of three cases involving full removal of the talon cusps during raising the periodontal flap to facilitate orthodontic treatment and to overcome occlusal interferences, followed by partial pulpotomy and follow‐up in periods ranging from 6 to 24 months [25]. In another case reports of anterior teeth with labial talon cusps, composite reconstruction and staged cuspal grinding of the labial talon cusp on the upper central incisor [19, 47] and canine [20] and on the lower central incisor to achieve appropriate esthetic results [18]. Orthodontic treatment was used to close a space after extraction of a mandibular left central incisor with a talon cusp for esthetic purposes [48]. In this study, a conservative approach was employed to reduce pocket depth, eliminate occlusal interference, and correct malocclusion. The treatment is aimed at gradually reducing the size and reshaping the form of the maxillary geminated central incisors, as well as decreasing the size of the talon cusp periodically. Following the reshaping of the central incisors and gradual reduction of the talon cusp, the periodontal pocket depth returned to the normal range for the gingival sulcus. Additionally, orthodontic treatment was planned to align the anterior teeth and relieve crowding.

These studies suggest that the anatomy of the talon cusp itself can cause occlusal interference, which may lead to periodontal tissue damage secondary to occlusal trauma. [18, 34, 47, 48]. In this case, the talon cusp was treated by eliminating the occlusal interference through selective grinding of its enamel surface until the patient was comfortable with occlusion and the interference resolved [49]. Sequential reduction of the talon cusp stimulates a pulpal response and the formation of tertiary dentin. Approximately 1–1.5 mm should be removed every 6–8 weeks [25]. It has been observed that reducing the full length of the talon cusp gradually, rather than just the incisal tip, promotes greater reparative dentin formation since most odontoblasts are located along the length of the cusp [25]. Fluoride should be applied after each odontoplasty session to minimize the risk of hypersensitivity [25, 49]. Sequential reduction of talon cusp induces a pulpal reaction and tertiary dentin formation; every 8–6 weeks, 1.5–1 mm was removed [29].

According to periodontal treatment protocols [32], managing periodontal pockets begins with the elimination of the causative factor in this case. The periodontal condition was associated with plaque retention resulting from the anatomical alteration created by the talon cusp rather than the cusp itself. Therefore, periodontal pocket management should start by reducing or removing the talon cusp. If the pocket depth does not decrease after 1 month, conventional periodontal therapy such as scaling and root planning should be initiated, followed by reassessment of pocket depth after 4 weeks [50]. This report describes a single rare case of bilateral geminated maxillary central incisors associated with a Type I talon cusp; therefore, the findings should be interpreted with caution and cannot be generalized to all patients with similar dental anomalies. Although a favorable clinical, periodontal, and functional outcome was observed following conservative multidisciplinary management, the relatively short follow‐up period limits assessment of the long‐term stability of the treatment outcomes, pulpal health, and periodontal status. In addition, the absence of histological evaluation restricts confirmation of the internal tissue characteristics of the talon cusp and geminated teeth. Further studies with larger case series and longer follow‐up periods are needed to better understand the etiology, periodontal implications, and long‐term prognosis of these uncommon dental anomalies.

## 4. Conclusion

Geminated teeth with a prominent talon cusp can result in a range of clinical complications. In this case, successful rehabilitation of the anterior maxillary geminated incisors with a talon cusp was accomplished through a multidisciplinary approach. Early diagnosis and timely intervention are essential to minimize potential complications and improve patient outcomes.

## Author Contributions

**Mohammed Sultan Al-Ak′hali:** conceptualization, methodology, investigation, data curation, writing – original draft, writing – review & editing, supervision. **Reem Hassan Kelani:** investigation, data curation, literature review, writing – review & editing. **Rahaf Ahmed Halawi:** data curation, literature review, visualization, writing – review & editing. **Hani Saeed Alakhali:** investigation, clinical data collection, management. **Atyaf Fassal Abueishah:** data curation, literature review, visualization, writing – review & editing. **Syed Wali Peeran:** conceptualization, methodology, supervision, validation, writing – review & editing.

## Funding

This study was supported by the Jazan University (10.13039/100009388; JU‐RSP2026).

## Disclosure

All authors have read and approved the final version of the manuscript. Mohammed Sultan Al‐Ak′hali had full access to all of the data in this study and takes complete responsibility for the integrity of the data and the accuracy of the data analysis.

## Conflicts of Interest

The authors declare no conflicts of interest.

## Data Availability

The data that support the findings of this study are available on request from the corresponding author. The data are not publicly available due to privacy or ethical restrictions.
